# Epigenetic Activation of Antibacterial Property of an Endophytic *Streptomyces coelicolor* Strain AZRA 37 and Identification of the Induced Protein Using MALDI TOF MS/MS

**DOI:** 10.1371/journal.pone.0147876

**Published:** 2016-02-04

**Authors:** Jitendra Kumar, Vijay K. Sharma, Dheeraj K. Singh, Ashish Mishra, Surendra K. Gond, Satish K. Verma, Anuj Kumar, Ravindra Nath Kharwar

**Affiliations:** 1 Mycopathology and Microbial Technology Laboratory, Department of Botany, Banaras Hindu University, Varanasi, 221005, India; 2 Dept of Botany, Visva-Bharati, Shantiniketan, Bolpur, India; 3 Dept of Botany, Budhha PG College, Kushinagar, India; National University of Ireland - Galway, IRELAND

## Abstract

The endophytic *Streptomyces coelicolor* strain AZRA 37 was isolated from the surface sterilized root of *Azadirachta indica* A. Juss., commonly known as neem plant in India. Since only a few reports are available regarding epigenetic modulations of microbial entities, *S*. *coelicolor* was treated with different concentrations of 5-azacytidine for this purpose and evaluated for its antibacterial potential against five human pathogenic bacteria (*Aeromonas hydrophila* IMS/GN11, *Enterococcus faecalis* IMS/GN7, *Salmonella typhi MTCC* 3216, *Shigella flexneri* ATCC 12022 and *Staphylococcus aureus* ATCC 25923). The crude extract obtained from cultures treated with 25 μM concentration of 5-azacytidine, was found effective against all five pathogenic bacteria tested while the untreated control was only active against 3 pathogenic bacteria. HPLC analysis of crude compounds from treated cultures showed a greater number of compounds than that of the control. Extraction of whole cell protein and its SDS PAGE analysis showed an additional major protein band in 25 μM 5-azacytidine treated culture and MALDI TOF MS/MS analysis revealed that this protein belongs to the porin family.

## Introduction

The emergence of antibiotic resistance in pathogenic microbes and prevalence of potent mutated strains, have created an alarming situation for both humans and crop plants. Since, natural bioactive compounds are known to control pathogens, there is an urgent need for continuous and rigorous search for novel natural products from different sources including plants, microorganisms and organisms inhabiting to unique niches. For millennia, human beings have been using plants and their products for the treatment of many diseases, but this approach has certain limitations such as biodiversity loss, high cost and intensive labour. Microorganisms may be the best alternative to this endeavour as a large amount of microbial biomass can be easily produced on a large scale. In this context it deserves mention that microbial natural products have been the major source of novel drugs for pharmaceutical industries for the last five decades, and many new reports clearly indicate that novel molecules with potential therapeutic applications are still waiting to be discovered, especially from actinomycetes [1]. Among all bioactive metabolites that have already been reported from microorganisms, the contribution of actinomycetes to this total is approximately half [2].

Soil has been a major habitat for actinomycetes, but recent studies indicate that these microbes are also abundantly present inside plant tissues as endophytes, and there is every possibility of discovery of novel strains and potential bioactive compounds from these organisms [3]. Endophytes play a major role in plant community health by providing resistance to hosts against diverse biotic and abiotic stresses [4, 5]. *Frankia*, a nitrogen-fixing microorganism was the first identified endophytic actinobacterium [6]. Various novel endophytic actinomycetes such as *Kribbella endophytica*, *Promicromonospora endophytica* [7, 8], *Allonocardiopsis opalescens* [9] and *Nocardia artemisiae* [10], have been reported from the surface sterilized tissues of different plants. From endophytic *Streptomyces* sp. (NRRL30566), antibiotic compound kakadumycins have been reported with wide spectrum antimicrobial activity [11]. Bafilomycins, a family of poleyene macrolides containing a 16-members lactone ring antibiotics, are produced by a variety of *Streptomycetes*, with different types of biological activities, such as antitumor, antifungal, antiparasitic and immunosuppressant [12].

*Azadirachta indica* (family Meliaceae) commonly known as neem, is the most useful folk medicinal plant in India, and almost all parts of the plant are useful for the treatment of different ailments. Isolation of over 400 bioactive compounds has been reported from various parts of ‘neem’ while only 32 compounds are reported from neem’s endophyte. This difference may be due to considerably fewer studies done on *A*. *indica* endophytes. Though fragmentary reports attest the work on fungal endophytes of neem and some other useful plants, more serious efforts are required to explore the diversity and potential of actinobacterial endophytes [13, 14, 15, 16, 17]. Interestingly, for the first time, Verma et al. have isolated 55 different isolates of actinomycetes from neem plant and screened them for their antibacterial and antifungal activities. They reported *Streptomyces* to be the dominant species followed by *Streptosporangium*, *Microbispora*, *Streptoverticillium*, *Sacchromonospora* sp., and *Nocardia* [3]. Quin et al. reported 2174 actinobacterial isolates from medicinal plants of tropical rain forests of Xishuangbanna (China) and these isolates were categorized into 46 taxa out of which 19 were considered novel using different modern taxonomic tools [18]. In another study, 123 endophytic actinomycetes were isolated from tropical plants at different locations of Papua New Guinae that were identified on 16S rRNA genes sequences and biosynthetic potential was assessed using putative polyketide synthase (PKS) and nonribosomal peptide synthetase (NRPS) genes [19]. It would be contextual to mention that *Sacchromonospora* sp., isolated from neem was used successfully to biosynthesize gold nanoparticles [20]. Recently, some interesting studies on diversity and biopotential of endophytic actinobacteria isolated from different medicinal plants have been conducted at different locations of India [21, 22, 23, 24].

The concept of epigenetics was introduced by Waddington for the development of specific traits by interaction of genes and its environmental factors [25]. According to Casadesus and Low [26] “epigenetic regulation is the expression of gene that takes place without changes of DNA sequences that can be achieved by methylation of specific DNA sequences by DNA methyl- transferase (DNMT)”. Gene clusters corresponding to natural products, which are silent in bacteria, can be expressed by treatment with epigenetic modulators such as 5-azacytidine and procain that inhibit DNMT. Interaction of regulatory proteins with gene promoter regions of natural products cluster can be improved by DNA demethylation that increases gene expression [27]. Often, epigenetic modulators are used to isolate cryptic metabolites and/or to enhance the production of known secondary metabolites. In a study, 5-azacytidine (5azaC), a DNA methyl- transferase inhibitor, was applied to cultures of *Streptomyces antibioticus* ETH7451 where SDS-PAGE analysis of proteins showed it affects a limited number of systems, and also increased the production of the antibiotic rhodomycin [28].

For the detection of proteins, matrix-assisted laser desorption/ionization time of flight mass spectrometry (MALDI-TOF-MS) is considered a powerful tool [29, 30, 31]. This technique can be used for identification of single protein, whole cell proteins and also for rapid identification of whole bacteria. Holland et al. [32] first reported the successful identification of five bacteria through chemotaxonomy using MALDI-TOF MS analysis of whole cells. The aims of this study were to select a bioactive isolate of actinobacteria associated with *A*. *indica*, and identification of cryptic metabolites that enhance the isolation of antimicrobial rich compounds through epigenetic modulations.

## Materials and Methods

### Isolation of Endophytic Actinomycetes from *Azadirachta indica*

For isolating the endophytic actinomycetes, plant tissue samples were collected from the Botanical Garden of Banaras Hindu University, Varanasi, Uttar Pradesh, India. Tissue samples were washed in running tap water for the removal of soil particles and other contaminants. Approximately 2 g of air dried tissue samples were kept in 70% ethanol for 1 minute, in 4% sodium hypochlorite for 2 minutes and again in 70% ethanol for 10 seconds followed by three times washing in sterilized distilled water. Excess water and sterilant were allowed to evaporate in laminar air flow cabinet. Tissue samples were crushed in a sterilized mortar and pestle with 10 ml sterilized distilled water. A 0.2 ml of crushed sample along with water was taken and spread on previously prepared starch casein potassium nitrate agar (SCA) medium plates [33]. SCA medium was amended with 100 μg/ml cycloheximide to restrict fungal growth. Plates were incubated at 28 ± 2°C for one month. After emergence, actinomycetes colonies were transferred to fresh SCA plates to obtaining pure isolates.

### Identification of Isolate AZRA 37

Isolated culture was identified by the DNA sequencing method. Total genomic DNA was isolated from an endophytic actinomycete by the slightly modified Pospiech and Neumann method [34]. Amplification of 16S rDNA was carried out by polymerase chain reaction (PCR) using a thermal cycler (Mycycler, BioRad). The PCR was carried out with approximately 100 ng of pure genomic DNA. The forward primer PA (AGA GTT TGA TCC TGG CTC AG) and the reverse primer PH (AAG GAG GTG ATC CAG CCG CA), were used for amplification of nearly entire gene. The amplification reactions were performed in a 50 μl volume, by mixing 2 μl of template DNA with 2 μl of each primer, 0.66 μl of (3u/μl) *Taq* polymerase (Genei, India), 1 μl dNTPs (10 μM each dATP, dCTP, dTTP and dGTP) (Genei, India), PCR buffer (10X) 5 μl and 37.34 μl MQ water for each reaction mixture. PCR amplified DNA was purified by HiYield PCR DNA mini kit from Real Biotech Corporation (RBC, India) through gel excision method. Purified DNA was sequenced by Amnion Biosciences Pvt. Ltd, India.

### Treatment of *Streptomyces coelicolor* Strain AZRA 37 with 5-Azacytidine

A 200 ml of starch casein potassium nitrate broth (SCB) medium was taken in five Erlenmeyer flasks (500 ml). Each flask was coded as A1 to A5 and amended with 0.5 μM to 250 μM concentration of 5-azacytidine. The control flask was not amended with 5-azacytidine. Freshly grown culture of AZRA 37 was inoculated in each flask and incubated at 28 ± 2°C in BOD incubator (NSW Calton, India) for one month.

### Extraction of Secondary Metabolites and Screening of Antibacterial Capacity

After one month of proper incubation, the cultures were filtered through Whatman no. 1 filter paper and supernatant was taken for extraction of secondary metabolites while the biomass kept for protein isolation. Equal volumes of ethyl acetate and supernatant both were taken in separating flask and mixed thoroughly for 15 minutes. The ethyl acetate phase was collected in new flask and this process was repeated thrice. Crude compounds containing ethyl acetate was evaporated in a rotatory evaporator (Ika, Germany). Concentrated crude compounds were taken out and kept in a fresh tube for drying in fume hood. Collected crude compounds were weighed and dissolved in methanol for final concentration of 100 μg/ μl. Antibacterial activity of crude compounds was evaluated by the modified Bauer-Kirby method [35]. Each sterile paper disc was impregnated with 10 μl methanolic extract and allowed to dryness at room temperature. Five human pathogenic bacteria (*Aeromonas hydrophila* IMS/GN11, *Enterococcus faecalis* IMS/GN7, *Salmonella typhi* MTCC 3216, *Staphylococcus aureus* ATCC 25923 and *Shigella flexneri* ATCC 12022) were taken for antibacterial test. Lawns of above bacteria were prepared on Mueller- Hinton agar (MHA) plates. Paper discs, each containing 1 mg crude compound were put on the surface of MHA plates, one paper disc impregnated with pure methanol was kept as control. All MHA plates were incubated at 37°C for overnight. Inhibition zones that formed around each paper disc were measured. Each test was done in three replicates.

Minimum inhibitory concentration (MIC), was calculated by MTT assay in 96 well microtitre plates. For the MTT assay, all compounds from different treatments of 5-azacytidine, were dissolved in methanol at 100 μg/μl concentration. Different concentration of dissolved compounds from 10 μg to 100 μg, were put into microtitre plate. Methanol was allowed to evaporate from the well. A 200 μl of Mulluer Hinton broth (MHB) was added into microtiter plate containing the compounds. Two μl test pathogenic bacterial culture (10^6^cells/ml) was inoculated in MHB containing microtitre plates and incubated overnight at 37°C. Further after incubation, 13.5 μl of MTT solution (5 mg/ml) was added into each well and incubated at 37°C for 30 min. Yellow color showed positive antibacterial activity while purple color showed negative activity. The minimum concentration at which yellow color maintained was recorded as the MIC.

### HPLC Analysis of Crude Compounds

HPLC analysis of crude compounds was done by RP- C18 Photodiode Array Detectors (PDA) with injection volume of 20 μl. The flow rate was 0.50 ml/min. Acetonitrile was the main mobile solvent along with double distilled water. Elution of compounds started with 15% acetonitrile reached up to 100% in 40 minutes that was held for 5 minutes, and again it came down to 15% acetonitrile in 8 minutes and finally it was held for 5 minutes. The samples and mobile phase were filtered through 0.2 μm nylon membrane filter before applying into the column. All samples were analyzed at 254 nm wavelength.

### Extraction and SDS PAGE Analysis of Proteins

Biomass of treated cultures was used for the extraction of proteins. The two grams pellet of the culture was suspended in 10 ml of 0.1 M phosphate- buffer saline (PBS). Cell disruption was done by pestle and mortar with liquid nitrogen and centrifuged at 13,000 rpm for 10 minutes at 4°C. The supernatant was collected and proteins were precipitated with the help of chilled acetone by mixing in 1:4 supernatant and acetone ratio and centrifugated at 13,000 rpm for 10 minutes at 4°C. The supernatant was discarded, and the pellet was kept at room temperature for complete evaporation of remaining acetone. Dried protein samples were redissolved in a minimum amount of PBS, collected in a fresh tube and stored at -20°C until further processing. For PAGE analysis, an equal volume of protein samples and sample buffer (Tris-HCl-62μM, SDS-2%, Glycerol-10%, β-mercaptoethanol-5%, EDTA 0.1%, Bromophenol blue 0.1%) were mixed, boiled for 3 minutes, centrifuged at 10,000 rpm for 10 minutes. Ten μl of each sample was run on 12% vertical SDS PAGE gel. After completion of run, gel was fixed in fixing solution (Methanol 50%, Acetic acid glacial 10%) for at least 30 minutes, afterward protein bands were visualized by silver staining.

### In Gel Trypsin Digestion and MALDI TOF MS/MS Analysis of the Induced Protein

The selected protein bands were excised with a clean blade and placed in 1.5 ml micro centrifuge tube. One hundred μl of washing solution (100μM ammonium bicarbonate and acetonitrile in equal amount) was added and incubated for 30 minutes with intermittent vortexing. The solvent was removed and the process was repeated 3–4 times. One hundred μl of acetonitrile was added and shaken for 3 minutes until the gel became white, then the solvent was removed and the gel was allowed for complete air dry. Protein was reduced with 75 μl each of 10 μM ditheothreitol (MP Biomedicals Pvt. Ltd., India) and 100 μM of ammonium bicarbonate at 50°C for 30 minutes. After cooling at room temperature, 50 μl each of 50 μM iodoacetamide (Serva electrophoresis GmbH, Heidelberg, Germany) and 100 μM ammonium bicarbonate were added, protein was alkylated for 30 minutes at room temperature in dark. Alkylation solution was discarded and gel was washed with 200 μl of washing solution for 10 minutes. The gel was dehydrated with acetonitrile for 5 minutes. For digestion of protein, 20 μl of trypsin (Amresco, Solon Ohio, USA) solution (25 ng/μl in 25 μM ammonium bicarbonate) was added and incubated for 16 hour at 37°C. Trypsin solution was transferred into fresh microcentrifuge tube. Peptides were extracted by addition of 30 μl of extraction solution (1:1 ratio of 1.0% trifluoroacetic acid and acetonitrile), and vortexed for 5 minutes. The supernatant was collected and kept with the previously collected trypsin solution. This step was repeated 3 times and the collected supernatant was evaporated using a vacuum concentrator until 5–6 μl sample is left.

The tripsinized peptide sample was subjected to MALDI TOF MS/MS analysis followed by homology search using MASCOT on a commercial basis from Interdisciplinary School of Life Sciences (ISLS), Banaras Hindu University, Varanasi, India. The trypsinized peptide sample was mixed with matrix (α-cyano-4-hydroxy cinnamic acid), spotted on ground steel plate and subjected to MALDI-TOF/MS (Bruker Daltonics) for mass spectrometric identification.

## Results

### Isolation and Identification of Endophytic Actinomycetes

**Fig 1:**
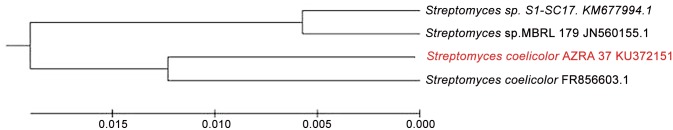
16S rDNA sequence based phylogenetic tree showing closest relatives of *Streptomyces coelicolor* strain AZRA 37 (accession KU372151) from NCBI GenBank database.

In this study, an endophytic actinomycete was isolated from the surface sterilized roots of *Azadirachta indica* (neem plant). On the basis of 16S rRNA gene sequencing, it was identified as *Streptomyces coelicolor* with Genbank accession number KU372151 (Fig 1, S1 File).

### Antibacterial Activity and Minimum Inhibitory Concentration of Treated Cultures

**Fig 2:**
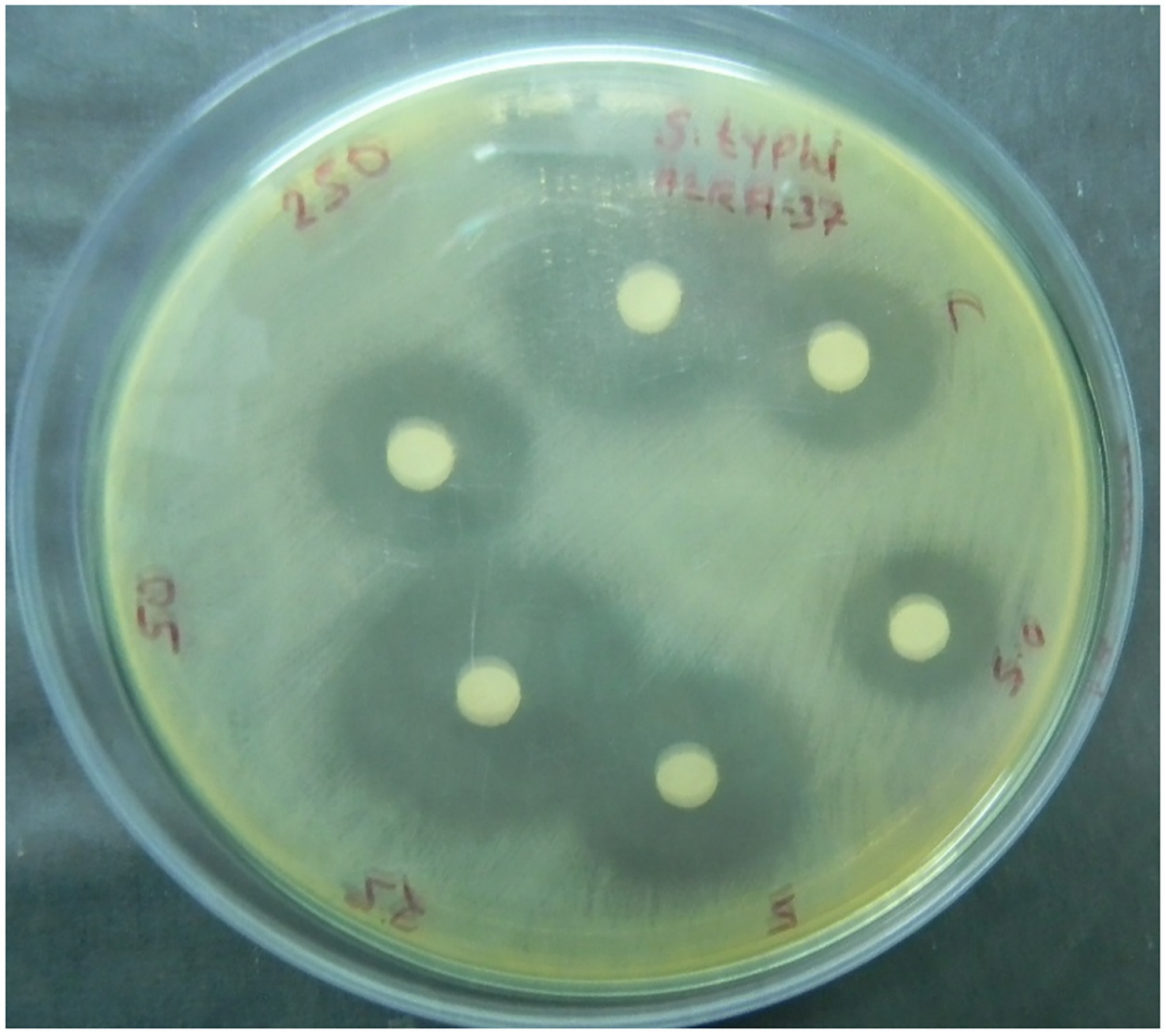
Antibacterial activity of crude compounds isolated from the control and treated (0.5, 5.0, 25, 50 and 250 μM 5-azacytidine) cultures of *Streptomyces coelicolor* strain AZRA 37.

Crude compounds when screened for antibacterial activity against five human pathogenic bacteria showed great susceptibility of *Aeromonas hydrophila* IMS/GN11, *Salmonella typhi* MTCC 3216 (Fig 2), and *Shigella flexneri* ATCC 12022. On the contrary, *Staphylococcus aureus* ATCC 25923 and *Enterococcus faecalis* IMS/GN7, were not at all susceptible.

In order to isolate cryptic metabolites, *Streptomyces coelicolor* was treated with variable concentration of the well known epigenetic modulator 5-azacytidine ranging from 0.5 μM to 250 μM. The crude compounds isolated from cultures isolates treated with different concentrations of 5-azacytidine were analyzed for antibacterial activity compared to the untreated isolate. It was observed that, 25 μM of 5-azacytidine treatment activated the antibacterial activity against two Gram positive bacteria *Staphylococcus aureus* ATCC 25923 and *Enterococcus faecalis* IMS/GN7, and also greatly enhanced the activity against all the three Gram negative bacteria (Table 1). Although concentrations of 50 μM and 250 μM of 5-azacytidine treated cultures were also active against tested bacteria but 25 μM was found to be the most effective.

**Table 1:** MIC of crude compounds of *Streptomyces coelicolor* strain AZRA 37 (accession KU372151) cultures treated with different concentrations of 5-azacytidine against 5 human pathogenic bacteria.

Concentration of 5-azacytidine (μM)	MIC value of crude compounds* against human pathogenic bacteria				
	*S*. *aureus*	*E*. *faecalis*	*A*. *hydrophila*	*S*. *typhi*	*S*. *flexneri*
Control	-	-	50	50	50
0.5 μM	-	-	50	60	50
5 μM	-	-	50	60	50
25 μM	60	60	40	40	40
0 μM	80	80	50	50	50
250 μM	80	80	70	70	60

*μg/ml

Minimum inhibitory concentration (MIC) of the crude compound was calculated by visual observation through MTT assay in micro titer plate. The crude compounds isolated from cultures treated with 25 μM 5-azacytidine showed the maximum antibacterial activity with 40 μg/ml of MIC against all the three Gram-negative bacteria and 60 μg/ml against the remaining two Gram-positive bacteria respectively. The crude compound from the untreated control was active against only three Gram-negative bacteria with 50 μg/ml of MIC. It was observed that, crude compounds from the treatment of 50 μM and 250 μM were also active against all the tested bacteria but their respective MIC values were higher than 25 μM and surprisingly, antibacterial activity was found in decreasing order with increasing concentration of 5- azacytidine (Table 1).

### HPLC Analysis of Crude Compounds

**Fig 3:**
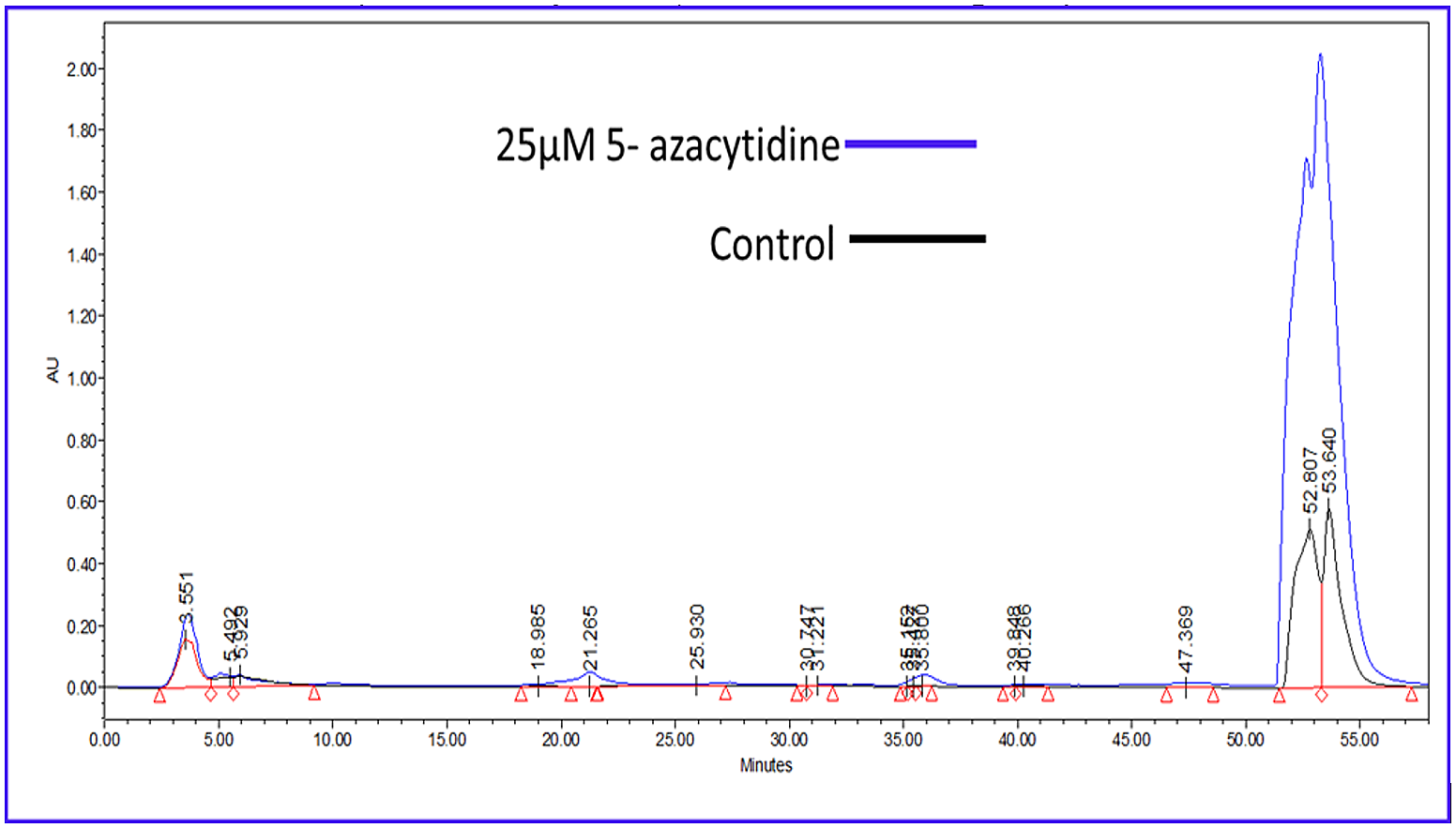
Reverse Phase HPLC chromatogram of 25 μM 5- azacytidine modulated crude product of isolate *Streptomyces coelicolor* strain AZRA 37 compared with the control showing increase in number and quantity of different compounds.

HPLC analysis of crude compounds of different treatments indicated that 25 μM treatment of 5-azacytidine activated the secretion of many cryptic compounds that were not observed in the untreated sample, however increased concentration of 5-azacytidine, decreased the concentration of cryptic compounds. This analysis clearly indicated that, crude compounds of the untreated control have produced five compounds while the treated culture (25 μM) secreted twelve (Fig 3).

### Protein Identification by MALDI TOF MS/MS

**Fig 4:**
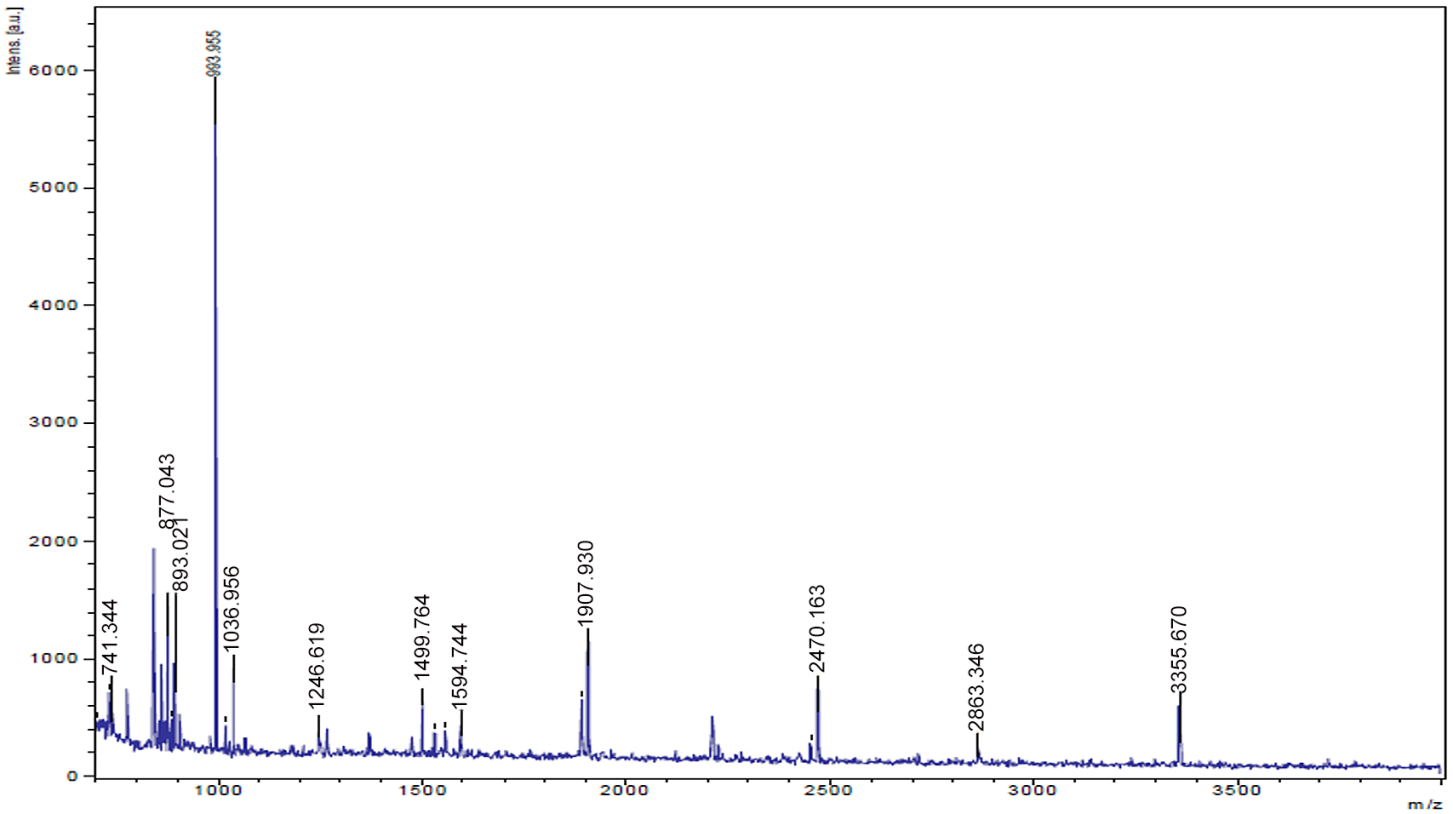
MS spectra of the induced 41 kDa porin protein after epigenetic modulation with 25 μM 5- azacytidine.

**Fig 5:**
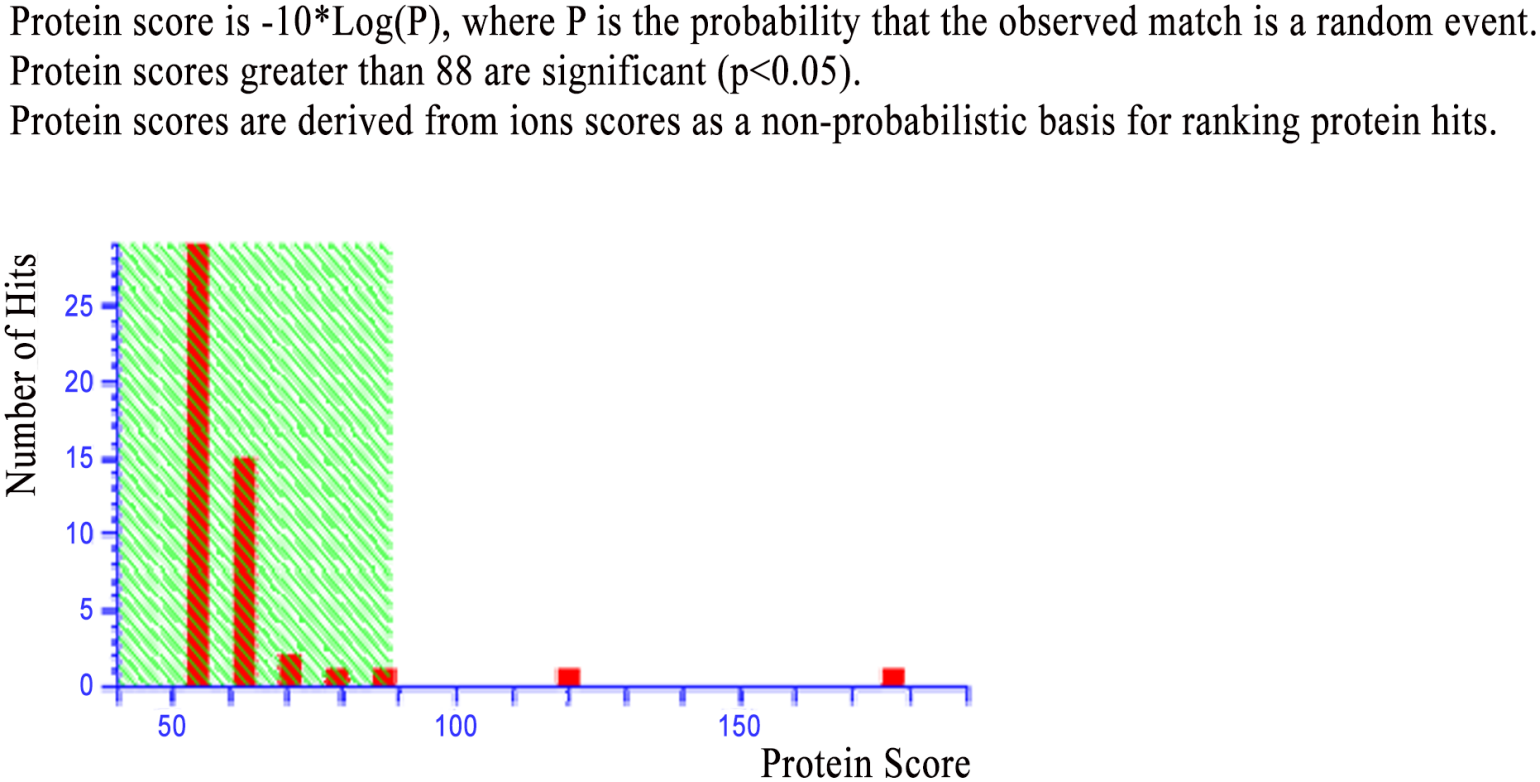
MS/MS Mascot search data of the induced porin protein.

A cryptic protein analyzed in treated sample using MALDI TOF MS/MS and MASCOT search showed close homology with porin family of proteins. MS data of the analyzed protein showed homology with porin family with total score of 126 and molecular mass of 40635 dalton (Fig 4, S1 Fig). MS/MS data showed same homology of porin family with total score of 177 and molecular mass of 40635 dalton. The MALDI-TOF/MS spectra and Mascot search data of this protein is given in the supporting information (Fig 5).

## Discussion

Various medicinal preparations have been derived from neem plant in different parts of the world for centuries [36]. Neem plants harbor a various types and huge numbers of microbes inside their internal tissues and actinomycetes are among them. Endophytic actinomycetes are reliable sources of different kinds of bioactive compounds [3]. This study was a part of an endeavour to isolate bioactive actinomycetes that have antimicrobial properties. Since 96.0% of total host bioactive compounds have been isolated from leaf, flower, seed and stem compared to 4.0% shares of root tissues therefore, root may harbor more microbial species than the tissues produce higher numbers of bioactive compounds [19]. For this experiment, a root inhabiting actinomycetes isolate was selected from the neem plant. In a preliminary study, this isolate showed very good antibacterial activity against three human pathogenic bacteria, therefore it was processed for further analysis. After 16S rRNA gene sequencing and its identification using sequences in the NCBI data base, it was found that isolate belongs to species *Streptomyces coelicolor* (Fig 1, S1 File). This isolate was grown on SCB medium with different concentration of 5- azacytidine for epigenetic activation of genes responsible for production of cryptic metabolites that are not produced under normal culturing conditions. 5- azacytidine acts as a DNA methyl transferase inhibitor and DNA methylation is the best known mechanism used in bacteria for alteration of epigenome [26]. Variations in gene expression are caused by methylation near the promoter regions of genes [37]. Genome sequencing of actinomycetes clearly indicated that, they have larger genomes relative to other bacterial species and they can use 5–10% of coding sequence for production of mostly cryptic metabolites [38].

Preliminary screening of this isolate for antibacterial activity was positive against three human pathogenic bacteria, but after treatment with 5-azacytidine, it gave positive activity against five pathogenic bacteria (Table 1). MIC of the crude compounds treated with 25 μM of 5-azacytidine was quite impressive against all the three Gram-negative bacteria (*Aeromonas hydrophila* IMS/GN11, *Salmonella typhi* MTCC 3216 and *Shigella flexneri* ATCC 12022) while it was moderate against the remaining two Gram-positive bacteria (*Staphylococcus aureus* ATCC 25923 and *Enterococcus faecalis* IMS/GN7). From the above results, it is clear that presence of 5-azacytidine in the growth medium, certainly stimulated the secretion of some additional active compounds into medium which were not produced under conditions without 5-azacytidine and these compounds may be responsible for positive antibacterial activity against tested pathogenic bacteria. It clearly indicates that some of the cryptic (silent) genes or gene clusters get activated and ultimately leads to production of cryptic metabolites [39]. Beau et al. [40] treated a marine fungus *Leucostoma persoonii* with 5-azacytidine (50 μM) and found the enhanced production of cytosporones B (360%), C (80%) and E (890%), and unknown cytosporone R, cytosporone E that were active against methicillin-resistant *Staphylococcus aureus* (MRSA). In this study, different concentrations of 5-azacytidine (0.5 μM -250 μM) were used, but 25 μM was found to be enough for activation of cryptic metabolites in broth medium. HPLC analysis showed the difference in compounds present in treated and untreated cultures in terms of number. Only five compounds were present in untreated cultures whereas in the case of treated cultures (25 μM) twelve compounds were recorded (Fig 3). Most of the cryptic compounds in treated cultures were found between retention time of 25 minutes and 55 minutes and these additional compounds may be responsible for antibacterial activity against another two bacterial species that were not susceptible to the crude metabolites of untreated cultures (Fig 3 and Table 1). The present findings are supported by a previous study where *Bacillus pumilus* cultures treated with some epigenetic modulators (5-azacytidine, prednisone, procaine, suberohydroxamic acid, and trimethoprim) with variable concentrations stimulated the production of some other minor peaks compared to untreated cells [27].

During analysis of proteins, an extra protein band of 41 kda was observed in treated cultures (25μM). MALDI TOF MS/MS analysis and mascot search results confirmed this protein as porin. To study the specific role of porin protein for enhanced antibacterial activity, a more focused study is required, but we assume that it plays a role in transport of 5- azacytidine across plasma membranes either inside or outside cells and it might be the reason for activation of silent genes/or gene clusters in production of the seven additional compounds [41]. *Nocardia farcinica* an actinobacterium, has shown the cation-selective cell wall channels (porins); these may be responsible for the limited permeability of negatively charged antibiotics [41]. It would be contextual to mention that this additional protein was observed in one dimensional SDS PAGE analysis that cannot represent all additional/or novel proteins in treated cells, and therefore, 2D gel electrophoresis/or gel free analysis may certainly enhance our understanding about the induced proteins of *Streptomyces coelicolor* treated with 25 μM 5-azacytidine.

## Supporting Information

S1 Fig Mascot search results of the MS of the induced protein porin (DOI: 10.6084/m9.figshare.2063193). (TIF)

S1 File 16S rDNA sequence of *Streptomyces coelicolor* strain AZRA 37 (Accession no. KU372151) (DOI: 10.6084/m9.figshare.2063184). (DOC)
