# Supplementary figures and images for: Epigenetic Activation of Antibacterial Property of an Endophytic Streptomyces coelicolor Strain AZRA 37 and Identification of the Induced Protein Using MALDI TOF MS/MS
Source: PLoS One. 2016 Feb 4;11(2):e0147876. doi: 10.1371/journal.pone.0147876 (PMC4742224; doi:10.1371/journal.pone.0147876)

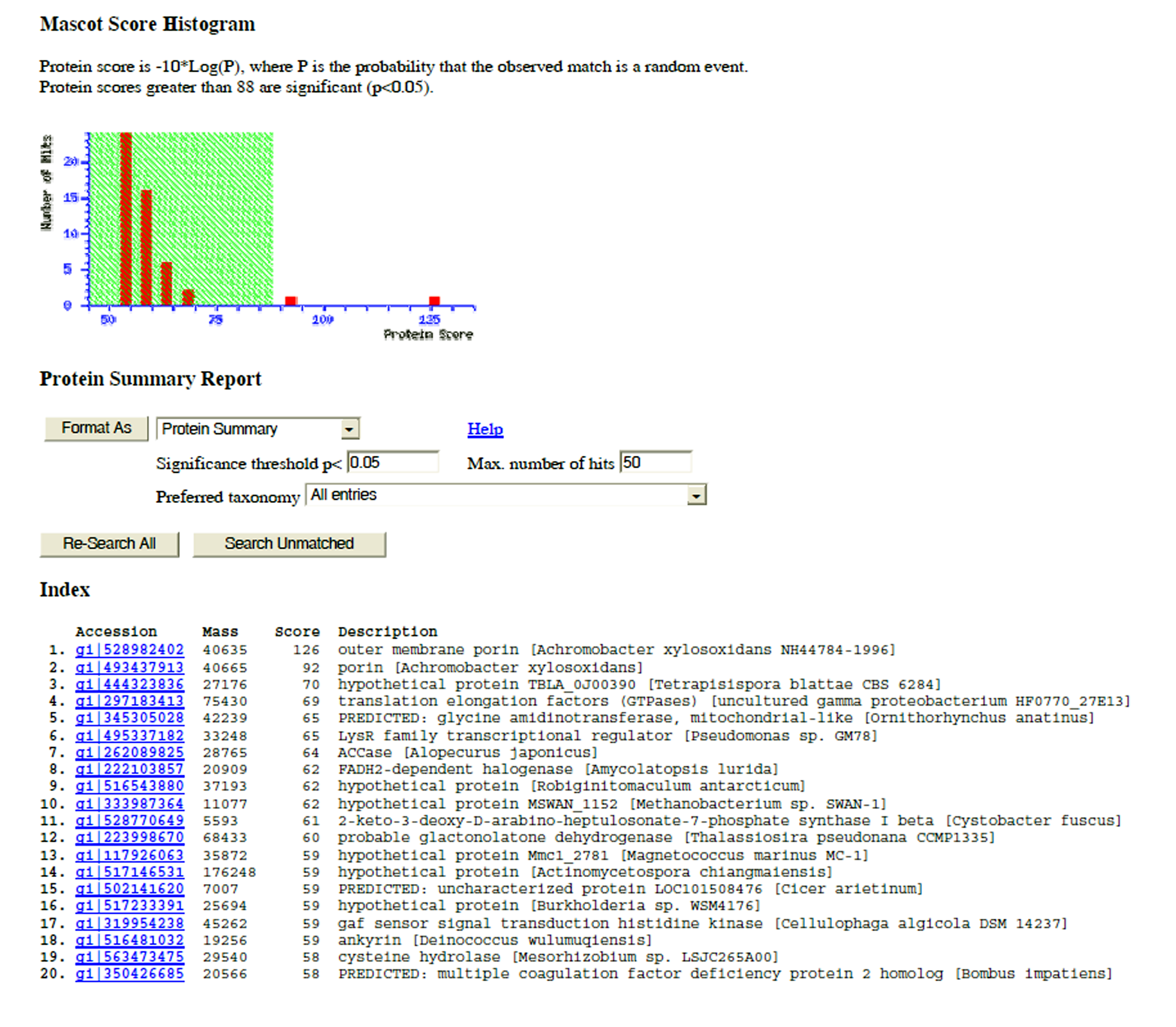

Supplement: S1 Fig — (TIF) [file pone.0147876.s001.tif]
